# Promising new tools to fight *Aedes* mosquitoes

**DOI:** 10.2471/BLT.16.020816

**Published:** 2016-08-01

## Abstract

Two new tools for suppressing *Aedes aegypti* mosquito populations have been recommended for pilot testing. Carefully designed trials will be needed to see whether they actually reduce disease as well. Andréia Azevedo Soares reports.

“When we heard that scientists were going to release mosquitoes in Tubiacanga a few years ago, we were afraid. ‘Why here?’ we wondered,” says Toni Salgado, a 52-year-old hairdresser who lives and works in the suburb of Rio de Janeiro in Brazil.

It is easy to understand Salgado's fear: *Aedes aegypti* is the main mosquito species that transmits the Zika, dengue, chikungunya and yellow fever viruses when it bites humans.

Communities affected by these diseases don't want more mosquitoes and for years they have tried in vain to get rid of them by covering containers or removing other objects that collect water – such as discarded bottles and used car tyres – to reduce mosquito-breeding grounds.

But the mosquitoes released in Rio de Janeiro, where the Olympic Games start on the 5th of this month, were not ordinary *Aedes* mosquitoes but ones that had been artificially infected with *Wolbachia –* a bacterium that stops dengue, chikungunya and Zika viruses from replicating inside their mosquito vectors.

**Figure:**
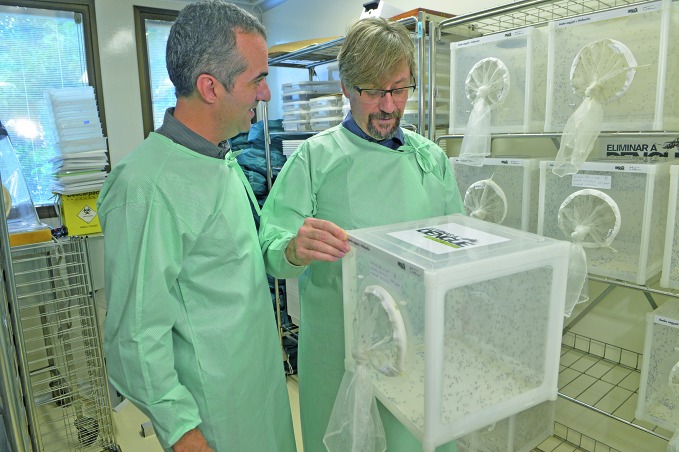
Luciano Moreira (left) and Scott O'Neill in the Fiocruz laboratory with a vat for breeding *Wolbachia*-infected mosquitoes

Eliminate Dengue Brazil

Scientists released 12 000 to 15 000 *Wolbachia*-infected mosquitoes every week for 20 weeks in this suburb of Rio de Janeiro from September 2014 and repeated this a year later.

When *Wolbachia*-infected mosquitoes mate with wild mosquitoes, they pass *Wolbachia* on to the next generation. If all goes to plan, most mosquitoes in Tubiacanga and Jurujuba, another neighbourhood involved in the study, will carry *Wolbachia* and be unable to transmit the three viruses to humans.

As Salgado recalls: “There was some hesitation in the beginning, in 2012, when the scientists first came here. But then we had meetings in which they explained to the community why this release was needed and residents agreed to participate. I am now proud to be part of it.”

“It’s a bone-breaker of a disease,” Salgado says, recalling how he suffered a bout of dengue before the study.

“We have gone through all the required small-scale field trials and the results are very promising in terms of the proliferation of *Aedes* infected with *Wolbachia*,” says Luciano Moreira, project leader at Oswaldo Cruz Foundation who coordinates the Brazilian part of the international, non-profit Eliminate Dengue research programme.

“Now we’re ready to scale-up the project. If we begin today, in three years we would have the capacity to cover a city like Rio de Janeiro.”

“Now we’re ready to scale-up the project. If we begin today, in three years we would have the capacity to cover a city like Rio de Janeiro.”

Luciano Moreira

Once the experiment is done on a large-scale – for example in Rio de Janeiro, a metropolitan area of more than 6 million people – scientists will be able to measure more accurately how much mass release of *Aedes* with *Wolbachia* actually reduces the incidence of dengue, Zika and chikungunya.

There are an estimated 390 million dengue infections globally every year, including 96 million with clinical symptoms.

Now a Zika epidemic across Latin America is raising international concerns about the virus’s association with birth defects such as microcephaly and neurological conditions, while chikungunya is on the rise too.

*Wolbachia* is naturally present in more than 60% of insect species, but *Aedes* is not one of them.

Scott O'Neill, a professor at the Monash University in Melbourne, Australia, leads the Eliminate Dengue research programme.

He and his team used microscopic needles to collect *Wolbachia* from fruit flies and inject the bacteria into the *Aedes* mosquito eggs. After “many thousands” of attempts, they succeeded.

“Imagine taking a knitting needle and poking it into a balloon. Next, you have to remove the needle without popping the balloon. That pretty well sums up the process of infecting mosquito eggs with *Wolbachia*,” O'Neill wrote in *Scientific American* last year.

After almost a decade of research, O'Neill's team managed to rear mosquitoes carrying *Wolbachia* in the laboratory. The first *Wolbachia*-infected *Aedes* were released in 2011 in Cairns, a small coastal city in Queensland, Australia.

O’Neill’s team did a similar experiment in Townsville, 346 km to the south, in October 2014 and other members of the research consortium have done similar field trials in Colombia, Indonesia and Viet Nam.

*Wolbachia*-infected *Aedes aegypti* was one of five promising new tools to reduce mosquito populations discussed at an emergency meeting of the World Health Organization (WHO) Vector Control Advisory Group in March in Geneva.

The meeting was held a month after WHO declared the Zika epidemic an international public health emergency.

At that meeting, experts from the Vector Control Advisory Group reviewed four other new tools: transgenic mosquitoes called Oxitec OX513A, vector traps, the sterile insect technique and the attractive toxic sugar bait.

They recommended the pilot deployment of two contrasting approaches: *Wolbachia*-infected *Aedes aegypti* mosquitoes and Oxitec transgenic mosquitoes to see whether the latter reduces mosquito populations when released on a large scale.

While the *Wolbachia* approach aims to make *Aedes* mosquito populations less harmful to human health, the Oxitec approach seeks to reduce the size of these populations.

**Figure:**
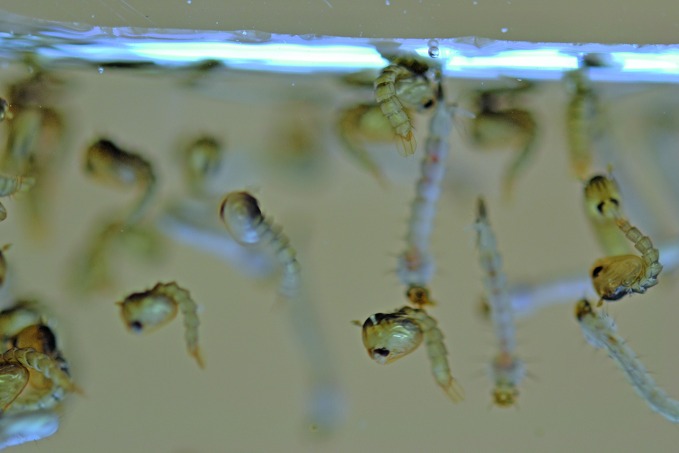
Oxitec (OX513A) mosquito larvae and pupae

Oxitec

Scientists at Oxitec, a subsidiary of the biotechnology company Intrexon based in England, developed the genetically modified mosquitoes back in 2002. These mosquitoes have a gene that prevents their offspring from surviving to maturity.

Only males are released, so there is no effect on disease transmission because only females bite humans. Once free, they mate with wild females and pass this self-limiting gene on to future generations.

According to Oxitec, five small-scale trials in Brazil, the Cayman Islands and Panama led to a reduction of more than 90% in mosquito populations.

Oxitec is awaiting approval from the United States Food and Drug Administration to do a large-scale trial near Key West in the southern state of Florida.

The company has been deploying genetically modified mosquitoes in Piracicaba in the Brazilian state of São Paulo in collaboration with the city authorities since April last year. 

“In January, we reported interim results already showing an 82% reduction in wild mosquito larvae,” says Simon Warner, chief scientific officer at Oxitec.

There are plans to increase the production of Oxitec mosquitos. “We are building a facility in Piracicaba that will be ready by the third quarter this year … to protect a population of 300 000 people,” Warner says. 

While both *Wolbachia*-infected and Oxitec mosquitoes have shown promise in terms of reducing or suppressing *Aedes aegypti* mosquito populations, it has yet to be proven that these interventions result in a reduction of the associated vector-borne diseases in humans. 

Without this evidence, it is impossible to fight epidemics of dengue, Zika and chikungunya effectively. Such evidence is now needed for these two promising new tools, but is also lacking when it comes to traditional methods of controlling mosquito populations. 

A systematic review published in March in *PLOS Neglected Tropical Diseases* entitled: “Is dengue vector control deficient in effectiveness or evidence?” concluded that “we do not have a clear understanding of which of the available interventions actually work, when they succeed or might work best” in terms of reducing dengue. 

“It is important to stress that lack of evidence does not mean lack of effectiveness,” says co-author Philip McCall, professor of medical entomology at the Liverpool School of Tropical Medicine in England.

“We need more randomized controlled trials to evaluate the most promising approaches to help affected countries design better evidence-based vector control strategies for reducing the disease burden,” McCall says. 

To support these countries members of the WHO Vector Control Advisory Group are preparing a manual on how best to design such studies. The manual will be released later this year.

At their meeting in March, the group stressed that some measures to protect humans from mosquitoes work if implemented well.

“The large-scale use of insecticide-treated bednets (ITNs) and indoor residual spraying have contributed significantly to the decline of malaria, for example,” says Dr Abraham Mnzava, coordinator of entomology and vector control in the Global Malaria Programme department.

However, ITNs are of limited use against *Aedes* because these mosquitoes bite during the day. Traditional approaches to tackling *Aedes* mosquitoes, such as fogging and space-spraying with insecticides and larvicides in household water storage containers, have been deployed for decades while dengue epidemics and resistance to insecticides have continued to grow.

 “New tools to reduce mosquito populations are needed, but these traditional methods are currently the only ones available and should, therefore, be promoted,” says Dr Raman Velayudhan, coordinator of vector ecology and management in the Department of Neglected Tropical Diseases at WHO headquarters.

“The lack of evidence that traditional approaches to vector control actually reduce human disease is one of the reasons why we say dengue is a neglected disease,” Velayudhan says, adding: “This may change in the next few years with the pilot deployment of these two promising new tools against the *Aedes aegypti* mosquito and carefully designed studies. This way we can rigorously monitor and evaluate not only whether these tools suppress mosquito populations – but also stop the spread of disease as well.”

